# Evolution of the Global Use of Unsafe Medical Injections, 2000–2010

**DOI:** 10.1371/journal.pone.0080948

**Published:** 2013-12-04

**Authors:** Jacques Pépin, Claire Nour Abou Chakra, Eric Pépin, Vincent Nault

**Affiliations:** Department of Microbiology and Infectious Diseases, Université de Sherbrooke, Sherbrooke, Québec, Canada; Vanderbilt University, United States of America

## Abstract

**Objective:**

Since 1999, substantial efforts have been made by the international community to reduce the risks associated with unsafe injections, through ministries of health, international donors, the World Health Organization and the Safe Injection Global Network. The present study attempted to measure the progress, or lack thereof, made over the 2000–2010 decade in reducing unsafe injections in ten regions of the world corresponding to developing and transitional economies.

**Methods:**

Data about the number of injections per person per year and the proportion of re-use of syringes and needles were obtained for 2010, mainly from population surveys, and compared with previous estimates for 2000 which had used various sources of information including injection safety assessments, population surveys and published studies on injection practices.

**Results:**

From 2000 to 2010, in developing countries and transitional economies, the average number of injections per person per year decreased from 3.40 to 2.88, while the proportion of re-use of injection devices dropped from 39.8% to 5.5%. Combining both factors the number of unsafe injections per person per year decreased from 1.35 to 0.16. Even if substantial progress has been made, the Eastern Mediterranean region remains problematic, with 0.57 unsafe injections per person per year. In sub-Saharan Africa and Latin America, people now receive on average only 0.04–0.05 unsafe injections per year.

**Conclusion:**

Substantial progress has been made in reducing the number of unsafe injections in developing countries and transitional economies, essentially through a reduction in the re-use of injection devices. In some regions, elimination of unsafe injections might become a reasonable goal.

## Introduction

The World Health Organization (WHO) defined a safe injection as one which does not harm the recipient, does not expose the provider to any avoidable risk and does not result in waste that is dangerous for the community [1]. Unsafe injections are linked to overuse of injections for mild conditions where oral drugs would be as effective (in Pakistan for instance, more than 90% of injections are thought to be unnecessary [2]), and to the re-use of injection equipment on several patients without adequate sterilization. Such re-use carries a risk of transmission of blood-borne viruses and represents the overwhelming majority of ‘unsafe healthcare injections’ or simply ‘unsafe injections’. In the year 2000, WHO estimated that unsafe healthcare injections accounted for 5% of HIV infections, 32% of hepatitis B virus (HBV) infections and 40% of hepatitis C virus (HCV) infections acquired in developing and transitional countries [3]–[5].

Since then, substantial efforts have been made by the international community, under the leadership of the Safe Injection Global Network (SIGN), to reduce the number of unsafe injections worldwide, so as to avoid the iatrogenic transmission of blood-borne viruses [1], [6], [7]. Its three core strategies are to promote behaviour change among patients and healthcare workers aiming to reduce unnecessary injections and ensure safe practices, to increase the availability of necessary and good quality injection devices and supplies, and to properly manage sharps waste. The present study addressed the first of the above-mentioned strategies and attempted to document the impacts of these efforts, by measuring the relative reduction or increase, from 2000 to 2010, in unsafe injections in various regions of the world.

For the sake of comparisons, the regions defined in the 2000 Global Burden of Diseases (GBD) study were used [3]–[5]. As for the year 2000, four high-income regions where unsafe injections were thought to be uncommon were excluded (North America/Cuba, Western Europe, Japan/Australia/New Zealand and other developed countries mostly in the Middle East). The ten regions of interest are, along with their acronyms, described in Table 1. These regions had been defined as per geography, standard WHO regions but also child mortality.

**Table 1:** Regions of the world (developing and transitional economies) as defined during the 2000 Global Burden of Diseases study.

**AFR D**	Algeria, Angola, Benin, Burkina Faso, Cameroon, Cape Verde, Chad, Comoros, Equatorial Guinea, Gabon, Gambia, Ghana, Guinea, Guinea-Bissau, Liberia, Madagascar, Mali, Mauritania, Mauritius, Niger, Nigeria, Sao Tome and Principe, Senegal, Seychelles, Sierra Leone, Togo
**AFR E**	Botswana, Burundi, Central African Republic, Congo, Côte d’Ivoire, Democratic Republic of the Congo, Eritrea, Ethiopia, Kenya, Lesotho, Malawi, Mozambique, Namibia, Rwanda, South Africa, Swaziland, Uganda, Tanzania, Zambia, Zimbabwe
**AMR B**	Antigua and Barbuda, Argentina, Bahamas, Barbados, Belize, Brazil, Chile, Colombia, Costa Rica, Dominica, Dominican Republic, El Salvador, Grenada, Guyana, Honduras, Jamaica, Mexico, Panama, Paraguay, Saint Kitts and Nevis, Saint Lucia, Saint Vincent and the Grenadines, Suriname, Trinidad and Tobago, Uruguay, Venezuela
**AMR D**	Bolivia, Ecuador, Guatemala, Haiti, Nicaragua, Peru
**EMR D**	Afghanistan, Djibouti, Egypt, Iraq, Morocco, Pakistan, Somalia, Sudan, Yemen
**EUR B**	Albania, Armenia, Azerbaijan, Bosnia and Herzegovina, Bulgaria, Georgia, Kyrgyzstan, Poland, Romania, Slovakia, Tajikistan, Macedonia, Turkey, Turkmenistan, Uzbekistan, Yugoslavia
**EUR C**	Belarus, Estonia, Hungary, Kazakhstan, Latvia, Lithuania, Republic of Moldova, Russian Federation, Ukraine
**SEAR B**	Indonesia, Sri Lanka, Thailand.
**SEAR D**	Bangladesh, Bhutan, Democratic People’s Republic of Korea, India, Maldives, Myanmar, Nepal
**WPR B**	Cambodia, China, Cook Islands, Fiji, Kiribati, Lao, Malaysia, Marshall Islands, Micronesia, Mongolia, Nauru, Niue, Palau, Papua New Guinea, Philippines, Republic of Korea, Samoa, Solomon Islands, Tonga, Tuvalu, Vanuatu, Viet Nam

## Methods

### Measurements for the year 2000

The annual number of injections per year per person (‘n’) and the proportion of re-use (‘p_r_’) had been estimated by WHO using the tools then available [3]–[5], [8], [9]. From these data, the annual number of unsafe injections per year per person (‘n_u_’) can be calculated as the product of ‘n’ and ‘p_r_’. At the time, the Demographic and Health Surveys (DHS) did not collect information on injection practices. For the estimation of ‘p_r_’, WHO had relied on studies of injections practices through standardized injection safety surveys in 10 African countries and Kyrgyzstan (observations in ≈80 randomly selected healthcare facilities in each country), on non-standardized surveys in Pakistan, India, China and Indonesia (observations in a convenience sample of health facilities), on back-calculations from relative risks (Egypt and Moldova), on extrapolations and on the combination of several methods [5]. These had been fetched from published literature, unpublished WHO reports, and SIGN reports. For the estimation of ‘n’, sources of information included population-based injection frequency surveys (14 countries) and other types of population-based data using the WHO guide for rapid assessment of injection practices (6 countries) [3]–[5], [9]. Further details are available in Table S1.

### Measurements for 2010

As data on injections were not available for each calendar year, the 2010 estimates used information generated as close as possible to this date, the limits being 2005 to 2011 (similar rounding had been used for 2000). Within each region, data from as many countries as possible were collected and regional estimates were calculated after weighting by total population size for the countries where estimates were available [10].

All DHS reports corresponding to the years 2005–2011, available up to 1 August 2012, were reviewed [11]. DHS are administered to a very large and representative sample of the adult population (generally defined as 15–49 years old) of targeted countries. In countries where this information had been collected, the following measures were extracted from the reports: i) the average number of injections received during the last year (or, in some countries, over the last 6 months, which was then doubled); ii) the proportion of participants who claimed that their last injection had been made with a syringe and a needle coming from an unopened package (‘p_r_’ was defined as one minus that proportion, after excluding those who could not answer this question ). In countries where two DHS had been published during the period of observation, the most recent one was used. The DHS used for ‘2010’ corresponded to four surveys in 2005, seven in 2006, seven in 2007, five in 2008, seven in 2009, six in 2010 and four in 2011. Fourteen DHS were available for region AFR E, nine for AFR D, three each for AMR B, SEAR D and WPR B, two each for AMR D, EMR D, EUR B and EUR C, and none for SEAR B (Table 1). Details of the 40 DHS are available in Table S2.

In countries with DHS data, no attempt was made to locate information from other sources. For countries without DHS data, the SIGN posts (weekly electronic newsletter) and SIGN annual meeting reports for 2005–2011 were reviewed using as search terms: ‘frequency of injection’, ‘prevalence of injection’, ‘injection per year’, ‘non-sterile’, ‘re-use’, as well as the names of each country. For China, Indonesia, Sri Lanka and Thailand, given the dearth of information on these countries a review of published literature was also made through Medline. Ultimately, SIGN data were used for three countries (Kenya, China and Mongolia), while two peer-reviewed publications were used for one country (China) [12]–[13]. No data were available for the three countries that constitute region SEAR B.

These 2010 estimates of ‘n’ and ‘p_r_’ were then compared to the 2000 data to measure progress, or lack thereof, during that decade. In the 2000 initial measure, in two regions where this information was available, the population above the 90^th^ percentile for ‘n’ had been excluded [3]–[5]. Since the DHS data used for 2010 did not exclude such individuals, the ‘n’ for 2000 were recalculated no longer excluding these outliers.

## Results

Table 2 displays the evolution in the number of healthcare injections per person per year (‘n’). Over the 10 regions of interest, ‘n’ decreased by 15% (from 3.40 to 2.88). The steepest reduction in the annual number of injections was seen in EUR C, while two regions saw an increase.

**Table 2:** Number of healthcare injections per person per year (‘n’) in each region, 2000 and 2010.

Region	Mean ‘n’, 2000	Number of countries with data	Mean ‘n’, 2010	Number of countries with data	
**AFR D**	2.09	3	1.16	9	
**AFR E**	1.90	6	1.33	14	
**AMR B**	1.62	2	3.23	3	
**AMR D**	1.88	1	1.12	2	
**EMR D**	4.23	2	4.03	2	
**EUR B**	5.20	1	4.03	2	
**EUR C**	11.30	1	2.28	2	
**SEAR B**	1.96	2	NA	0	
**SEAR D**	3.92	1	1.93	3	
**WPR B**	2.30	1	4.18	3	
**Overall**	3.40	20	2.88	40	

NA: Not available

The proportion of re-use, ‘p_r_’, decreased in all but two regions (Table 3). Overall, ‘p_r_’ dropped from 39.8% to 5.5%, a relative reduction of 86%. Only in AMR B and EUR B did the proportion of re-use apparently increase.

**Table 3:** Proportion of re-use (‘p_r_’) during healthcare injections in each region, 2000 and 2010.

Region	Mean ‘p_r_’2000	Number of countries with data	Mean ‘p_r_’2010	Number of countries with data	
**AFR D**	0.190	5	0.033	9	
**AFR E**	0.170	5	0.035	12	
**AMR B**	0.012	0	0.016	3	
**AMR D**	0.110	0	0.048	2	
**EMR D**	0.700	2	0.141	2	
**EUR B**	0.012	1	0.061	2	
**EUR C**	0.110	1	0.055	2	
**SEAR B**	0.300	1	NA	0	
**SEAR D**	0.750	1	0.071	3	
**WPR B**	0.300	1	0.040	2	
**Overall**	0.398	17	0.055	37	

NA: Not available.

Multiplying ‘n’ with ‘p_r_’, it can be seen in Table 4 that substantial progress was achieved in reducing the average number of unsafe injections per person per year (‘n_u_’) in seven regions, where this number decreased by at least 70%. In AMR B and EUR B, the number of unsafe injections has apparently increased.

**Table 4:** Number of unsafe healthcare injections per person per year (‘n_u_’) in each region, 2000 and 2010.

Region	Mean ‘n_u_’, 2000	Mean ‘n_u_’, 2010	
AFR D	0.40	0.04	
AFR E	0.32	0.05	
AMR B	0.02	0.05	
AMR D	0.21	0.05	
EMR D	2.96	0.57	
EUR B	0.07	0.25	
EUR C	1.37	0.13	
SEAR B	0.59	NA	
SEAR D	2.94	0.14	
WPR B	0.69	0.17	
Overall	1.35	0.16	

NA: Not available.

Thus, by and large, most of the progress was achieved through a reduction in the percentage of injections made with re-used equipment, while more modest gains were made through a reduction in the annual number of injections. Combining both factors the number of unsafe injections per person per year decreased by 88%, from 1.35 to 0.16.

Using the data in Tables 2 and 4 and population figures for each region [10], it can be calculated that in 2010 15.7 billion injections were made in the ten regions of interest, close to the 16 billion estimated for 2000 [5]: the reduction in the number of injections per capita was compensated by the population growth. In 2010, approximately 874 million unsafe injections were given throughout the world.

To estimate to what extent our measures of changes from 2000 to 2010 may have been biased by the use of different data collection methods, and to determine whether similar time trends were seen when two national surveys had been performed with the same method, additional data are provided as Supporting Information. Table S3 shows data from eight countries where ‘p_r_’ was estimated through both a DHS and an injection safety survey performed within 3 years of each other. With the exception of Ukraine and Uganda, the differences in ‘p_r_’ were less than 2.0%, suggesting that systematic biases between the two data collection methods were not very marked. Table S4 summarizes data from countries where two DHS with injection data were performed, typically within 5–6 years of each other. There were three countries where little change was seen in the mean number of injections per year (≤0.1), four countries where ‘n’ increased and four where it decreased. However, ‘p_r_’ decreased in five out of six countries, and there was little change (0.1%) in the other. Table S5 displays data from countries where ‘p_r_’ was estimated through two injection safety surveys, within 3–6 years of each other. The proportion of re-use decreased in all countries except South Africa and in those where ‘p_r_’ had been estimated to be zero in the first place. Although the number of countries in these tables is limited and the interval between surveys rather short, taken together these figures are compatible with a reduction in ‘p_r_’, but little or no change in ‘n’.

## Discussion

There were already estimates of the number of unsafe injections per person per year in the year 2000. What this study adds to the literature is a measurement of the same parameters in 2010, after more than 10 years of international efforts to reduce the infectious risks of unsafe injections.

The main finding is that the number of potentially unsafe injections has decreased by 88% between 2000 and 2010 in developing and transitional economies. This estimate was based on the best tools available, which were however imperfect and varied between the first and second measurement.

The data for the annual number of injections and the proportion of re-use may have been more robust for 2010 than for 2000, because the former was based on a higher number of countries than the latter. For 2010, DHS data were available for 40 countries (37 of them with an estimate of both ‘n’ and ‘p_r_’), while for 2000, 32 countries provided measures (but only 5 with estimates of both ‘n’ and ‘p_r_’) [3]–[5].

In 2010, DHS were the main sources of information, and data on ‘n’ may have been more reliable than before as large and representative samples of the population were interviewed in each country, especially in sub-Saharan Africa. Nevertheless, DHS are prone to information biases. Some patients may not notice whether the injection they are receiving is made with a new needle and syringe from an unopened package, or may not remember it well when interviewed months later, one among dozens of questions on various topics [11]. On the other hand, in 2000, most data on ‘p_r_’ came from small observational studies prone to another type of bias: healthcare workers who know that they are being observed may decrease their use of previously used needles and syringes [3]–[5]. Evidence from a limited number of countries (Table S3) suggests, however, a reasonable degree of concordance between the two types of measures [14].

The temporal changes in each region are much less robust than the global ones and must be interpreted with great caution because of the above-mentioned biases, but also because of the limited number of countries with data in some regions. For instance, the 2000 ‘n’ measures for AMR B was based on Brazil and Latino communities in the USA, while the 2010 measure was based on the Dominican Republic, Honduras and Guyana; the comparison and the apparent increase in injections might have been biased by the use of some data from the USA in 2000. The 2000 ‘n’ measure for EUR B was based only on Romania, while that for 2010 used data from Armenia and Azerbaijan. For both ‘n’ and ‘p’, measurements for EUR C in 2000 were derived from Moldova alone, while in 2010 Ukraine, ten times more populous, was added to the latter. In China, which represents four fifths of the population of WPR B, the measures of ‘n’ were based on surveys in selected regions which were not necessarily representative of the whole country.

DHS being generally carried out every 5 years, we had to use data collected between 2005 and 2011. If the reduction in unsafe injections continued during the interval between the year of the DHS and 2010, the mean number of unsafe injections in 2010 might have been overestimated, and the true reduction underestimated. Should the current study be repeated in the future, there will be many countries with two (or more) DHS measures of injection practices, where it will be possible to calculate the mean annual change in ‘n’, ‘p_r_’ and ‘n_u_’ and extrapolate to the years involved in the future comparison, assuming a linear trend. Such repeat measures, with the same method, may also show that some countries failed to improve their performance, and additional investments will need to be made in these jurisdictions. The inclusion of injection data within many DHS during the last decade represented by itself a substantial progress towards injections safety, as what is measured is more likely to be acted upon than what remains unknown.

Despite the above limitations, it seems likely that there was indeed a substantial decrease in the number of unsafe injections between 2000 and 2010, due to the concerted efforts of ministries of health, local health facilities, non-profit organizations, donors and SIGN. For the future, approaches targeting both a reduction in the overall number of injections and a reduction in the proportion of injections given with re-used syringes and needles are generally recommended, so as to maximise the impact of injection safety programmes in achieving rational and safe use of injections.

Over the last decade, the reduction in the overall number of injections per capita, safe and unsafe, was only 15%. In the few countries where two DHS were carried out (Table S4), albeit over a shorter interval (≈5 years), there was no evidence of a systematic reduction in the mean number of injections per year. This reflects the reality of medical practice in the developing world and transitional economies. Many injections are given in the private and informal sectors, where the quest for profit implies that health care providers do not respect recommendations from public health officials to avoid unnecessary injections, but rather prefer to satisfy requests made by patients, some of whom believe that injected drugs are more powerful than oral meds [15]. Furthermore, these beliefs about the superiority of injections can be shared by some healthcare workers themselves [5]. Information, education and communication efforts need to be maintained, targeting both the injection prescribers and the patients. Reducing the number of unnecessary injections is a long-term goal that requires the collaboration of all stakeholders and cannot be implemented as a vertical programme [16], [17].

There has been remarkable progress in reducing the proportion of injections made with re-used material. Similar trends can be seen in most countries where two surveys using the same methodology were conducted (Tables S4 and S5). In several regions of the world, 5% of injections or less are currently made with re-used syringes and needles (Table 3). It may be possible, during the upcoming decade, to eliminate completely unsafe injections from some parts of the world, eliminating at the same time injection-related HIV, HCV and HBV infections. Considering the data shown in Table 4, elimination of this risk could be a reasonable goal in sub-Saharan Africa and the Americas. Ideally, these countries should encourage the importation of syringes and needles which cannot be re-used, at least for outpatient services where the majority of injections are given.

The risk of transmission of blood-borne viruses from unsafe injections is not static. Even if its performance much improved, EMR D remains worrying given the high number of unsafe injections per capita in 2010 (more than three times the worldwide average, and up to 15 times higher than in the poorest regions of the world), and a near trebling of HIV prevalence over the last decade [18]. Already in 2000, that region represented one third of the injections-related HCV infections in the world [1]. In Egypt, where 14.7% of individuals aged between 15 and 59 years are HCV-seropositive, 14% of injections are made with re-used syringes and needles [19]. Along with EUR B, EMR D should be a priority region for SIGN and national authorities. It would seem wiser to anticipate this risk and act now rather than wait until the damage of large-scale iatrogenic HIV infections is documented.

## Supporting Information

Table S1 Countries with data used for the 2000 measurements.
(DOCX)

Table S2 Countries with data from Demographic and Health Surveys (DHS) used for the 2010 measurements.
(DOCX)

Table S3 Countries where the proportion of re-use (‘p_r_’) was measured within a three-year interval through both a Demographic and Health Survey^11^ and an injection safety survey.
(DOCX)

Table S4 Countries where two Demographic Health Surveys (DHS) were performed which included injection data.
(DOCX)

Table S5 Countries where two injection safety surveys were performed which measured the proportion of re-use (‘p_r_’).
(DOCX)
