# Supplementary material for: Evolution of the Global Use of Unsafe Medical Injections, 2000–2010
Source: PLoS One. 2013 Dec 4;8(12):e80948. doi: 10.1371/journal.pone.0080948 (PMC3851995; doi:10.1371/journal.pone.0080948)
Supplement: Table S1 — Countries with data used for the 2000 measurements. (DOCX) [file pone.0080948.s001.docx]

**Table S1.** Countries with data used for the 2000 measurements.^5^

| **Region** | **Countries with data on the number of injections per year** | **Countries with data on the proportion of re-use** |
| --- | --- | --- |
| **AFR D** | Guinea-Bissau, Cameroon, Nigeria | Burkina Faso, Chad, Gambia, Mauritania, Niger |
| **AFR E** | Burundi, Central African Republic, Côte d’Ivoire, Tanzania, Uganda, Zambia | Eritrea, Ethiopia, Swaziland, Zambia and Zimbabwe |
| **AMR B** | Brazil, Latino communities in USA | None |
| **AMR D** | Haiti | None |
| **EMR D** | Egypt, Pakistan | Egypt, Pakistan |
| **EUR B** | Romania | Kyrgyzstan |
| **EUR C** | Moldova | Moldova |
| **SEAR B** | Thailand, Indonesia | Indonesia |
| **SEAR D** | India | India |
| **WPR B** | China | China |
