# Supplementary material for: Evolution of the Global Use of Unsafe Medical Injections, 2000–2010
Source: PLoS One. 2013 Dec 4;8(12):e80948. doi: 10.1371/journal.pone.0080948 (PMC3851995; doi:10.1371/journal.pone.0080948)
Supplement: Table S2 — Countries with data from Demographic and Health Surveys (DHS) used for the 2010 measurements. (DOCX) [file pone.0080948.s002.docx]

**Table S2**. Countries with data from Demographic and Health Surveys (DHS) used for the 2010 measurements.^11^

|  |  | **Number of injections per year** | | | **Proportion of injections with new needle** | | | **Propor.**  **re-used** |  |
| --- | --- | --- | --- | --- | --- | --- | --- | --- | --- |
| **Country or Region** | **Year of DHS** | **Women** | **Men** | **Men + women** | **Women** | **Men** | **Men + women** | **Men + women** | **Non sterile injec. per**  **year** |
| **AFR D** |  |  |  |  |  |  |  |  |  |
| Benin | 2006 | 1.20 |  | 1.20 | 0.965 |  | 0.965 | 0.035 | 0.042 |
| Ghana | 2008 | 1.00 | 0.80 | 0.90 | 0.975 | 0.975 | 0.975 | 0.025 | 0.023 |
| Liberia | 2007 | 1.40 | 1.40 | 1.40 | 0.937 | 0.952 | 0.945 | 0.056 | 0.078 |
| Madagascar | 2008-2009 | 1.20 | 0.50 | 0.85 | 0.980 | 0.948 | 0.964 | 0.036 | 0.031 |
| Mali | 2006 | 0.80 |  | 0.80 | 0.958 |  | 0.958 | 0.042 | 0.034 |
| Nigeria | 2008 | 1.10 | 1.40 | 1.25 | 0.959 | 0.977 | 0.968 | 0.032 | 0.040 |
| Sao Tome | 2008-2009 | 1.10 | 1.30 | 1.20 | 0.979 | 0.981 | 0.980 | 0.020 | 0.024 |
| Senegal | 2010-2011 | 1.10 | 0.80 | 0.95 | 0.974 | 0.969 | 0.972 | 0.029 | 0.027 |
| Sierra Leone | 2008 | 1.60 | 2.20 | 1.90 | 0.958 | 0.931 | 0.945 | 0.056 | 0.105 |
| **AFR E** |  |  |  |  |  |  |  |  |  |
| Burundi | 2010 | 1.00 | 0.70 | 0.85 | 0.991 | 0.969 | 0.980 | 0.020 | 0.017 |
| Congo | 2005 | 14.00 | 12.80 | 13.40 | 0.941 | 0.970 | 0.956 | 0.045 | 0.596 |
| Cote d'Ivoire | 2005 | 0.90 | 0.80 | 0.85 | 0.921 | 0.941 | 0.931 | 0.069 | 0.059 |
| Ethiopia | 2011 | 1.60 | 1.10 | 1.35 | 0.975 | 0.976 | 0.976 | 0.025 | 0.033 |
| Lesotho | 2009 | 1.10 | 0.50 | 0.80 |  |  |  |  |  |
| Malawi | 2010 | 0.90 | 0.40 | 0.65 |  |  |  |  |  |
| Mozambique | 2009 | 0.70 | 0.40 | 0.55 | 0.915 | 0.931 | 0.923 | 0.077 | 0.042 |
| Namibia | 2006-2007 | 3.60 | 2.80 | 3.20 | 0.970 | 0.954 | 0.962 | 0.038 | 0.122 |
| Rwanda | 2010 | 1.50 | 0.80 | 1.15 | 0.988 | 0.992 | 0.990 | 0.010 | 0.012 |
| Swaziland | 2006-2007 | 1.10 | 0.70 | 0.90 | 0.964 | 0.933 | 0.949 | 0.052 | 0.046 |
| Uganda | 2006 | 2.00 | 1.50 | 1.75 | 0.975 | 0.963 | 0.969 | 0.031 | 0.054 |
| Tanzania | 2010 | 1.30 | 0.70 | 1.00 | 0.981 | 0.957 | 0.969 | 0.031 | 0.031 |
| Zambia | 2007 | 0.90 | 0.60 | 0.75 | 0.969 | 0.972 | 0.971 | 0.030 | 0.022 |
| Zimbabwe | 2010-2011 | 0.70 | 0.70 | 0.70 | 0.982 | 0.975 | 0.979 | 0.022 | 0.015 |
| **AMR B** |  |  |  |  |  |  |  |  |  |
| Dominican Republic | 2007 | 2.50 | 2.20 | 2.35 | 0.992 | 0.979 | 0.986 | 0.015 | 0.034 |
| Guyana | 2009 | 1.00 | 1.00 | 1.00 | 0.954 | 0.955 | 0.955 | 0.046 | 0.046 |
| Honduras | 2005-2006 | 4.60 |  | 4.60 | 0.984 |  | 0.984 | 0.016 | 0.074 |
| **AMR D** |  |  |  |  |  |  |  |  |  |
| Bolivia | 2008 | 1.70 |  | 1.70 | 0.930 |  | 0.930 | 0.070 | 0.119 |
| Haiti | 2005-2006 | 0.70 | 0.40 | 0.55 | 0.991 | 0.955 | 0.973 | 0.027 | 0.015 |
| **EMR D** |  |  |  |  |  |  |  |  |  |
| Egypt | 2008 | 2.40 | 1.00 | 1.70 | 0.845 | 0.870 | 0.857 | 0.143 | 0.246 |
| Pakistan | 2006-2007 | 5.10 |  | 5.10 | 0.860 |  | 0.860 | 0.140 | 0.714 |
| **EUR B** |  |  |  |  |  |  |  |  |  |
| Armenia | 2010 | 2.00 | 1.20 | 1.60 | 0.988 | 0.915 | 0.952 | 0.049 | 0.078 |
| Azerbaijan | 2006 | 7.30 | 2.40 | 4.85 | 0.943 | 0.926 | 0.935 | 0.066 | 0.318 |
| **EUR C** |  |  |  |  |  |  |  |  |  |
| Moldova | 2005 | 6.00 | 3.20 | 4.60 | 0.985 | 0.989 | 0.987 | 0.013 | 0.060 |
| Ukraine | 2007 | 2.50 | 1.70 | 2.10 | 0.969 | 0.914 | 0.942 | 0.059 | 0.123 |
| **SEAR D** |  |  |  |  |  |  |  |  |  |
| India | 2005-2006 | 2.10 | 1.80 | 1.95 | 0.912 | 0.943 | 0.928 | 0.073 | 0.141 |
| Maldives | 2009 | 4.70 | 2.60 | 3.65 | 0.931 | 0.932 | 0.932 | 0.069 | 0.250 |
| Nepal | 2011 | 1.10 | 1.30 | 1.20 | 0.978 | 0.987 | 0.983 | 0.018 | 0.021 |
| **WPR B** |  |  |  |  |  |  |  |  |  |
| Cambodia | 2010 | 3.30 | 1.80 | 2.55 | 0.982 | 0.976 | 0.979 | 0.021 | 0.054 |
| Samoa | 2009 | 0.30 | 0.40 | 0.35 |  |  |  |  |  |
| Viet Nam | 2005 | 1.60 | 1.30 | 1.45 | 0.946 | 0.968 | 0.957 | 0.043 | 0.062 |
| Timor-Leste | 2009-2010 | 0.80 | 0.20 | 0.50 | 0.982 | 0.979 | 0.981 | 0.020 | 0.010 |
|  |  |  |  |  |  |  |  |  |  |
