# Supplementary material for: Evolution of the Global Use of Unsafe Medical Injections, 2000–2010
Source: PLoS One. 2013 Dec 4;8(12):e80948. doi: 10.1371/journal.pone.0080948 (PMC3851995; doi:10.1371/journal.pone.0080948)
Supplement: Table S3 — Countries where the proportion of re-use (‘pr’) was measured within a three-year interval through both a Demographic and Health Survey11 and an injection safety survey. (DOCX) [file pone.0080948.s003.docx]

**Table S3**. Countries where the proportion of re-use (‘p_r_’) was measured within a three-year interval through both a Demographic and Health Survey^11^ and an injection safety survey.

|  | **Demographic and Health Survey** | | **Injection safety survey** | |
| --- | --- | --- | --- | --- |
| **Country** | **Year** | **‘p_r_’** | **Year** | **‘p_r_’**^a^ |
| Nigeria | 2008 | .032 | 2011 | .022 |
| Ukraine | 2007 | .059 | 2008 | .000 |
| Côte d'Ivoire | 2005 | .069 | 2004 | .060 |
| Ethiopia | 2011 | .025 | 2009 | .040 |
| Rwanda | 2010 | .010 | 2009 | .010 |
| Uganda | 2006 | .031 | 2003 | .150 |
| Tanzania | 2010 | .031 | 2009 | .050 |
| Nepal | 2011 | .018 | 2012 | .030 |

^a^ for curative injections
