# Supplementary material for: Evolution of the Global Use of Unsafe Medical Injections, 2000–2010
Source: PLoS One. 2013 Dec 4;8(12):e80948. doi: 10.1371/journal.pone.0080948 (PMC3851995; doi:10.1371/journal.pone.0080948)
Supplement: Table S4 — Countries where two Demographic Health Surveys (DHS) were performed which included injection data. (DOCX) [file pone.0080948.s004.docx]

**Table S4**. Countries where two Demographic Health Surveys (DHS) were performed which included injection data.^11^

|  | **First DHS** | | **Second DHS** | |
| --- | --- | --- | --- | --- |
| **Country** | **Year** | **Mean number of**  **injections per year** | **Year** | **Mean number of**  **injections per year** |
| Ethiopia | 2005 | 1.05 | 2011 | 1.35 |
| Malawi | 2004 | 0.55 | 2009 | 0.65 |
| Rwanda | 2005 | 2.55 | 2010 | 1.15 |
| Tanzania | 2004 | 1.10 | 2010 | 1.00 |
| Uganda | 2004 | 2.20 | 2011 | 1.85 |
| Zimbabwe | 2005–06 | 0.50 | 2010–11 | 0.70 |
| Lesotho | 2004 | 0.40 | 2009 | 0.80 |
| Cambodia | 2005 | 1.85 | 2010 | 2.55 |
| Armenia | 2005 | 1.85 | 2010 | 1.60 |
| Haiti | 2005–06 | 0.55 | 2012 | 0.60 |
| Honduras, women only | 2005–06 | 4.60 | 2011–12 | 1.90 |
|  |  | **Proportion of**  **re-use** |  | **Proportion of**  **re-use** |
| Ethiopia | 2005 | .062 | 2011 | .025 |
| Rwanda | 2005 | .080 | 2010 | .010 |
| Tanzania | 2004 | .030 | 2010 | .031 |
| Zimbabwe | 2005–06 | .073 | 2010–11 | .022 |
| Cambodia | 2005 | .029 | 2010 | .021 |
| Haiti | 2005–06 | .027 | 2012 | .018 |
