# Supplementary material for: Evolution of the Global Use of Unsafe Medical Injections, 2000–2010
Source: PLoS One. 2013 Dec 4;8(12):e80948. doi: 10.1371/journal.pone.0080948 (PMC3851995; doi:10.1371/journal.pone.0080948)
Supplement: Table S5 — Countries where two injection safety surveys were performed which measured the proportion of re-use (‘pr’). (DOCX) [file pone.0080948.s005.docx]

**Table S5**. Countries where two injection safety surveys were performed which measured the proportion of re-use (‘p_r_’).

|  | **First injection safety survey** | | **Second injection safety survey** | |
| --- | --- | --- | --- | --- |
| **Country** | **Year** | **‘p_r_’** | **Year** | **‘p_r_’** |
| Kenya | 2004 | .030 | 2009 | .010 |
| South Africa | 2004 | .050 | 2009 | .075 |
| Gambia | 2005 | .020 | 2009 | .010 |
| Burkina | 2000 | .040 | 2003 | .010 |
| Tajikistan | 2004 | .050 | 2007 | .010 |
| Georgia | 2002 | .000 | 2007 | .000 |
| Ukraine | 2002 | .000 | 2008 | .000 |
| Armenia | 2003 | .000 | 2006 | .000 |
| Haiti | 2004 | .050 | 2009 | .020 |
